# Similarity and Potential Relation Between Periimplantitis and Rheumatoid Arthritis on Transcriptomic Level: Results of a Bioinformatics Study

**DOI:** 10.3389/fimmu.2021.702661

**Published:** 2021-11-09

**Authors:** Shiyi Li, Changqing Zhou, Yongqian Xu, Yujia Wang, Lijiao Li, George Pelekos, Dirk Ziebolz, Gerhard Schmalz, Zeman Qin

**Affiliations:** ^1^ Department of Implantology, Stomatological Hospital, Southern Medical University, Guangzhou, China; ^2^ Department of Emergency, The Eighth Affiliated Hospital, Sun Yat-sen University, Shenzhen, China; ^3^ South Campus Outpatient Clinic, Sun Yat-sen Memorial Hospital, Sun Yat-sen University, Guangzhou, China; ^4^ Department of Implantology, Department of General Dentistry, Sun Yat-sen Memorial Hospital, Sun Yat-sen University, Guangzhou, China; ^5^ Central Sterile Supply Department, Sun Yat-sen Memorial Hospital, Sun Yat-sen University, Guangzhou, China; ^6^ Division of Periodontology & Implant Dentistry, Faculty of Dentistry, The University of Hong Kong, Sai Ying Pun, Hong Kong, SAR China; ^7^ Department of Cariology, Endodontology and Periodontology, University of Leipzig, Leipzig, Germany

**Keywords:** periimplantitis, rheumatoid arthritis, bioinformatics, cross-talk genes, CD14, FCGR2B

## Abstract

**Background:**

This bioinformatics study aimed to reveal potential cross-talk genes, related pathways, and transcription factors between periimplantitis and rheumatoid arthritis (RA).

**Methods:**

The datasets GSE33774 (seven periimplantitis and eight control samples) and GSE106090 (six periimplantitis and six control samples) were included from the National Center for Biotechnology Information (NCBI) Gene Expression Omnibus (GEO). A differential expression analysis (*p* < 0.05 and |logFC (fold change)| ≥ 1) and a functional enrichment analysis (*p* < 0.05) were performed. Based on this, a protein–protein interaction (PPI) network was constructed by Cytoscape. RA-related genes were extracted from DisGeNET database, and an overlap between periimplantitis-related genes and these RA-related genes was examined to identify potential cross-talk genes. Gene expression was merged between two datasets, and feature selection was performed by Recursive Feature Elimination (RFE) algorithm. For the feature selection cross-talk genes, support vector machine (SVM) models were constructed. The expression of these feature genes was determined from GSE93272 for RA. Finally, a network including cross-talk genes, related pathways, and transcription factors was constructed.

**Results:**

Periimplantitis datasets included 138 common differentially expressed genes (DEGs) including 101 up- and 37 downregulated DEGs. The PPI interwork of periimplantitis comprised 1,818 nodes and 2,517 edges. The RFE method selected six features, i.e., MERTK, CD14, MAPT, CCR1, C3AR1, and FCGR2B, which had the highest prediction. Out of these feature genes, *CD14* and *FCGR2B* were most highly expressed in periimplantitis and RA. The final activated pathway–gene network contained 181 nodes and 360 edges. Nuclear factor (NF) kappa B signaling pathway and osteoclast differentiation were identified as potentially relevant pathways.

**Conclusions:**

This current study revealed FCGR2B and CD14 as the most relevant potential cross-talk genes between RA and periimplantitis, which suggests a similarity between RA and periimplantitis and can serve as a theoretical basis for future research.

## Introduction

Rheumatoid arthritis (RA) is an auto-inflammatory disease, primarily affecting the joint cartilage and leading to pain and disability for the affected patients (1). Thereby, it is a rare disease, as approximately 1% of world’s population suffers from RA (2). It has been extensively shown in literature that RA can be related to inflammatory periodontal disease like periodontitis (3–5). Patients with RA show more severe periodontal inflammation and damage (6, 7); moreover, it is discussed controversially whether there is a causal relationship between periodontitis and RA (4, 5). Especially, an inflammatory dysbalance might connect RA with oral inflammation (8).

Periimplantitis is the inflammation of implant surrounding tissue alongside with loss of supporting bone (9). Periimplantitis affects approximately 18% of patients treated with dental implants (10). Although the basic etiology and pathology of periimplantitis appear similar as for periodontitis, the counterpart of periodontitis around dental implants is not mandatory (11, 12). Periimplant inflammation shows several histopathological differences against periodontitis, including a higher aggressiveness and bone loss (13). Especially due to the high importance of immune system and the partially autoimmune character of periimplantitis as well as potential genetic predisposition for inflammation (14–16), the relationship between periimplant inflammation and RA appears somewhat reasonable and of clinical interest. Available literature did not confirm an association between periimplantitis and RA (17, 18), yet. However, only one clinical study focused on clinical periimplantitis parameters and showed more pronounced marginal bone loss and bleeding at implants in RA individuals (19). Therefore, a similarity and the potential link between RA and periimplantitis remain a promising field of research.

Recently, the molecular mechanism, including genetics and epigenetics between oral and systemic diseases, has been successfully explored by bioinformatics analysis. So an interlink between periodontitis and Alzheimer’s disease, or between periimplantitis and type 2 diabetes mellitus, has been detected (20, 21). Accordingly, this current study used bioinformatics to reveal the cross-talk mechanisms between periimplantitis and RA at the transcriptomic level. Thereby, it was aimed to reveal potential cross-talk genes, related pathways, and transcription factors (TFs). It was hypothesized that cross-talk genes exist between RA and periimplantitis, supporting a similarity between these two diseases and thus an auto-inflammatory character of periimplant inflammation.

## Material and Methods

### Datasets and Study Design

**Figure 1:**
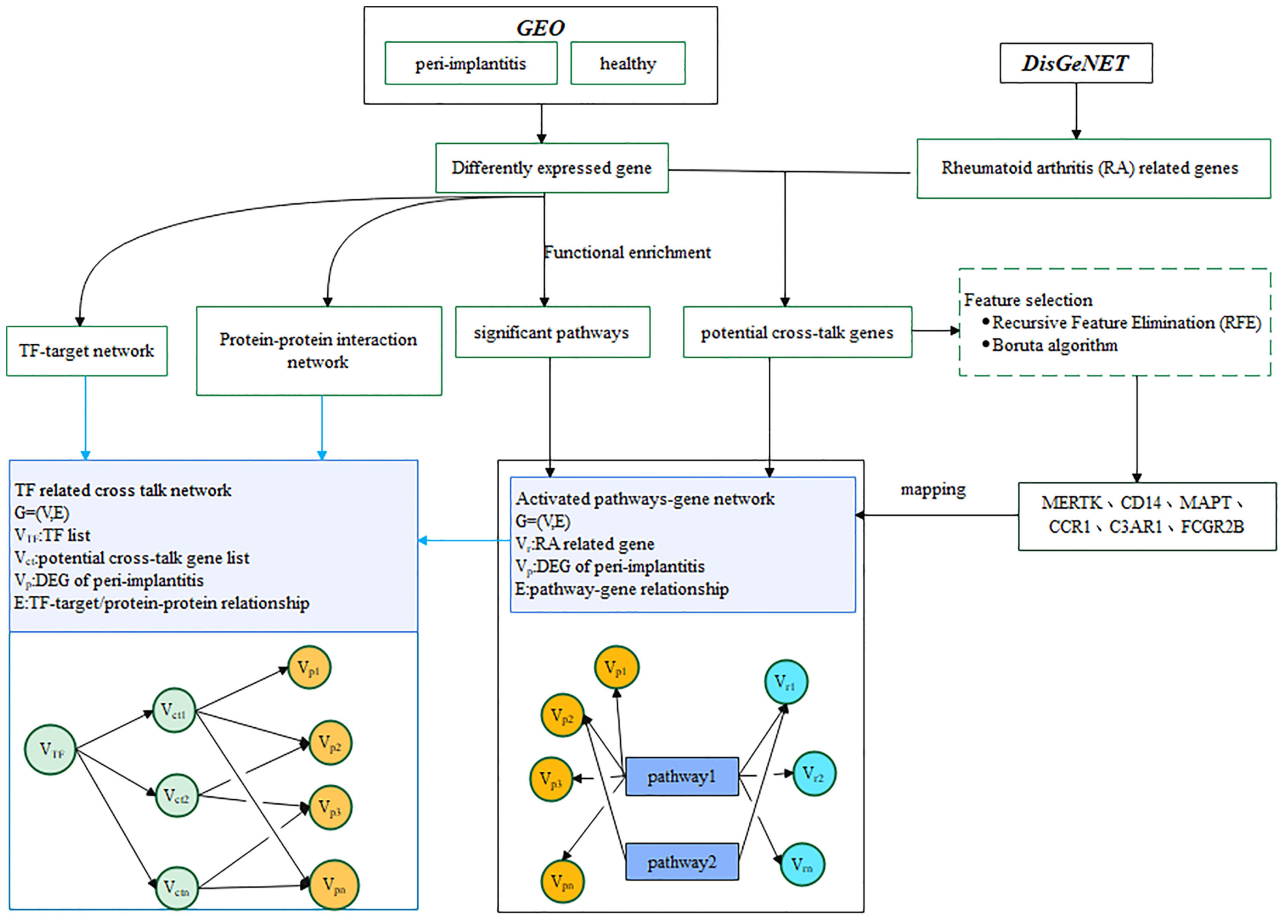
The framework of the current study.

Two datasets for periimplantitis were included, i.e., GSE33774 and GSE106090, from the National Center for Biotechnology Information (NCBI) Gene Expression Omnibus (GEO). The eligibility criteria were as follows: datasets that included established periimplantitis samples as the periimplantitis group and healthy gingival samples as the control group. Accordingly, eight controls and seven periimplantitis patients were included form GSE33774, while six periimplantitis tissue samples and six healthy periodontal tissue samples as the control group were obtained from GSE106090. The analytic workflow is shown in **Figure 1**.

### Differential Gene Expression Analysis

Differences between the periimplantitis and control groups for GSE33774 and GSE106090 were assessed using Linear Models for Microarray (limma) package in R project (version 3.0.1, http://www.r-project.org/). Genes with *p*-value <0.05 and |logFC (fold change)| ≥ 1 were regarded as significant differentially expressed genes (DEGs).The common up-/downregulated DEGs in GSE33774 and GSE106090 were used for the following analyses, and DEGs that were not common to all of the datasets were excluded. The applied “limma” analysis was performed based on a valid linear model (22). The limma package includes statistical methods that i) facilitate information using empirical Bayes methods to obtain posterior variance estimators, ii) incorporate observation weights (referring to the sample) for variations in data quality, iii) allow variance modeling to accommodate technical or biological heterogeneity, and iv) include pre-processing such as variance stabilization to reduce noise. These methods all help to improve inference at both gene and gene-set level in small experiments.

### Functional Enrichment Analysis

Gene Ontology (GO) biological process and pathway enrichment analysis of up-/downregulated periimplantitis-related DEGs were performed with clusterProfiler of R Bioconductor packages (https://bioconductor.org/packages/release/bioc/html/clusterProfiler.html). Thereby, the enriched biological functions of all DEGs, irrespective of whether they were up- or downregulated, were examined. The enriched function with *p*-value <0.05 was significant. Based on this analysis, the top 35 GO biological processes, which were ranked by the *p*-value in ascending order, and all the significant pathways were selected.

The clusterProfiler package offers a gene classification method to classify genes based on their projection at a specific level of the GO corpus and provides functions to calculate enrichment test for GO terms and Kyoto Encyclopedia of Genes and Genomes (KEGG) pathways based on hypergeometric distribution. To prevent high false discovery rate (FDR) in multiple testing, values are also estimated for FDR control. Furthermore, clusterProfiler supplies a function to automatically calculate enriched functional categories of each gene clusters and provides several methods for visualization. The current analyses were performed with regard to previously published literature (23).

### Construction of Protein–Protein Interaction Network

Protein–protein interactions (PPIs) were extracted from BIOGRID (Biological General Repository for Interaction Datasets), HPRD (Human Protein Reference Database), DIP (Database of Interacting Proteins), MINT (Molecular INTeraction database), PINA (Protein Interaction Network Analysis), InnateDB (A knowledge resource for innate immunity interactions & pathways), and Instruct (3D protein interactome networks with structural resolution). The Cytoscape platform was used to construct the PPI network and analyze the network topological characteristics.

### Identification of Potential Cross-Talk Genes

RA-related genes were downloaded from the DisGeNET database (https://www.disgenet.org/home/). The potential cross-talk genes, which were RA-related genes and overlapped with the up- and downregulated periimplantitis-related DEGs, were identified. These cross-talk genes linking periimplantitis and RA were used for constructing a cross-talk gene-related PPI network.

### Transcription Factor Regulated Cross-Talk Genes

TF–target gene regulation pairs were downloaded from TRRUST, TRANSFAC, cGRNB, ORTI, and HTRIdb databases. According to the TF–target relationship, the periimplantitis-related TF–target pairs were extracted, and then the TF–target gene interaction network was built and visualized using Cytoscape software.

### Support Vector Machine Modeling Using Feature Selection Cross-Talk Genes

The gene expression of GSE33774 and GSE106090 was merged, and batch correction for the merged data was performed with the ComBat method of sva packages in R project. The batch effect was eliminated for the intersection gene expression profiles of the two datasets GSE33774 and GSE106090. It should be noted that the gene sample expression values obtained after the batch effect was eliminated were different from the original gene expression values of the two datasets. Then, the resulting data were standardized using the Scale method obtained by R, and finally the support vector machine (SVM) model was established. Thereby, the original expression spectrum of the two datasets GSE33774 and GSE106090 was obtained and directly standardized with Scale. Subsequently, the expression values of the potential cross-talk genes from the merged data were determined. Finally, feature selection was performed by the Boruta algorithm in R project and the conventional Recursive Feature Elimination (RFE) algorithm.

Gene expression values of the screened feature genes that form the merged gene expression profile were extracted, and then SVM model with the gene expression value and sample type was built (periimplantitis or healthy). Firstly, the merged expression profile was normalized by scale method of R project. Subsequently, python scikit-learn package was used to perform grid search to find the best hyperparameters of the SVM model(s) using fivefold cross-validation (CV) and built SVM models. The merged data were divided into training sets and test sets, the ratio of which is 70%:30%. After the SVM model was built using the training sets, the prediction effectiveness of the model with the test sets was evaluated. Furthermore, the evaluation score for each sample was acquired with the decision_function of python. Finally, the prediction was assessed by receiver operating characteristic (ROC) curves with the pROC package and displayed using the ggplot2 package in R.

### Pathway Analysis of the Cross-Talk Genes

To find activated pathways, the significantly enriched pathways by the DEGs of periimplantitis were screened. For the significant pathways, the potential cross-talk pathways, which may be the bridge of RA disease and periimplantitis, were selected, and a directed network was created: G*=(V,E)*, where *V* stands for the gene sets in the pathway and *E* stands for the interactions between pathways and genes. The genes for each pathway were obtained. Then, the genes that are neither periimplantitis-related DEGs nor RA-related genes were removed. The pathway–gene cross-talk network was constructed by using the Cytoscape software. In the network G*=(V,E)*, Vr was the RA related genes and Vp was the DEGs of periimplantitis including the potential cross-talk genes.

In order to identify the functional TFs, which regulated the cross-talk in the activated pathways, the cross-talk genes in the pathway–gene pairs were extracted, and then the TFs that targeted the cross-talk genes was extracted. Furthermore, the periimplantitis-related DEGs in the pathway–gene pairs, which were interacted with cross-talk genes in the pathway–gene pairs, were selected. In the network, all DEGs in periimplantitis were denoted by Vp, TFs were denoted by V_TF_, and potential cross-talk genes were denoted by Vct. Finally, the relationship among TFs, cross-talk genes, and periimplantitis-related genes was constructed.

## Results

### Different Expressed Genes of Periimplantitis

The obtained DEGs from GSE33774 and GSE106090 are shown in **Table 1**. Sample characteristics are provided in **Supplementary Table 1**.

**Table 1:** The DEG number of GSE33774 and GSE106090.

Accession	Platforms	Periimplantitis samples	Healthy control samples	Total genes	Upregulated	Downregulated
GSE33774	GPL6244	7	8	23,304	158	66
GSE106090	GPL21827	6	6	32,763	2,092	2,238

DEG, differentially expressed gene.

**Figure 2:**
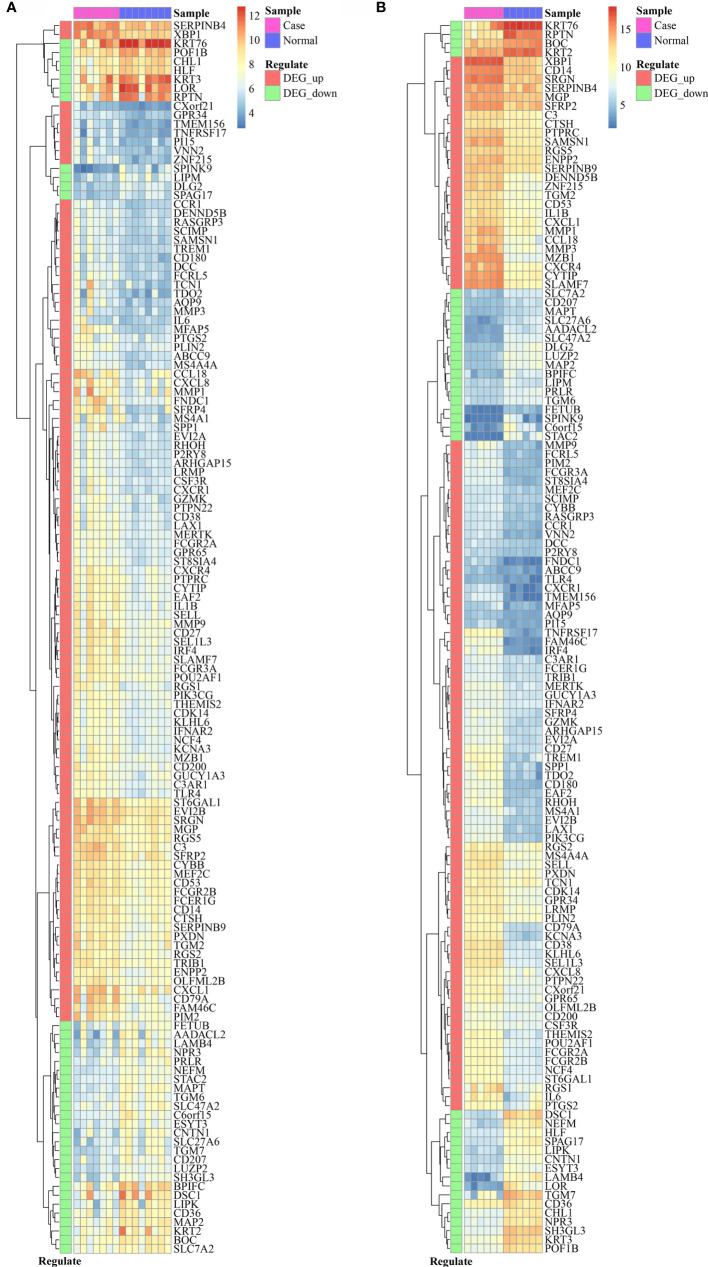
The expression level of 138 common DEGs in GSE33774 **(A)** and GSE106090 **(B)**. DEGs, differentially expressed genes.

The comparison of DEGs between GSE33774 and GSE106090 revealed 138 common DEGs including 101 upregulated and 37 downregulated genes. The expression levels of common DEGs are displayed in **Figures 2A, B**.

### Functional Enrichment

**Figure 3:**
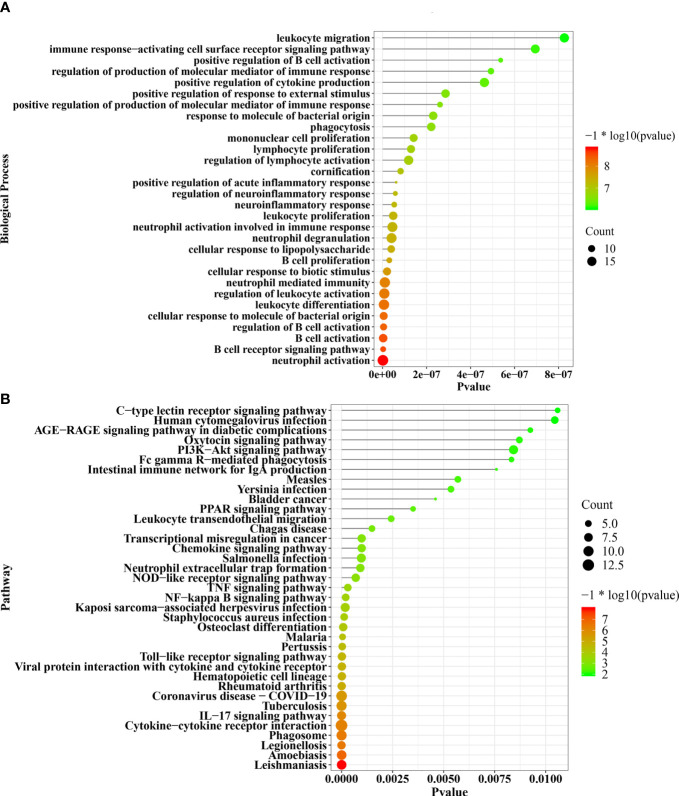
The significant enriched biological processes and pathways of 138 common DEGs **(A, B)**. DEGs, differentially expressed genes.

The DEGs significantly enriched in the biological processes were most strongly related to neutrophil activation, B-cell receptor signaling pathway, B-cell activation, and cellular response to molecule of bacterial origin (**Figure 3A**). The respective biological pathways in which DEGs are involved are shown in **Figure 3B**.

### Protein–Protein Interaction Network

**Figure 4:**
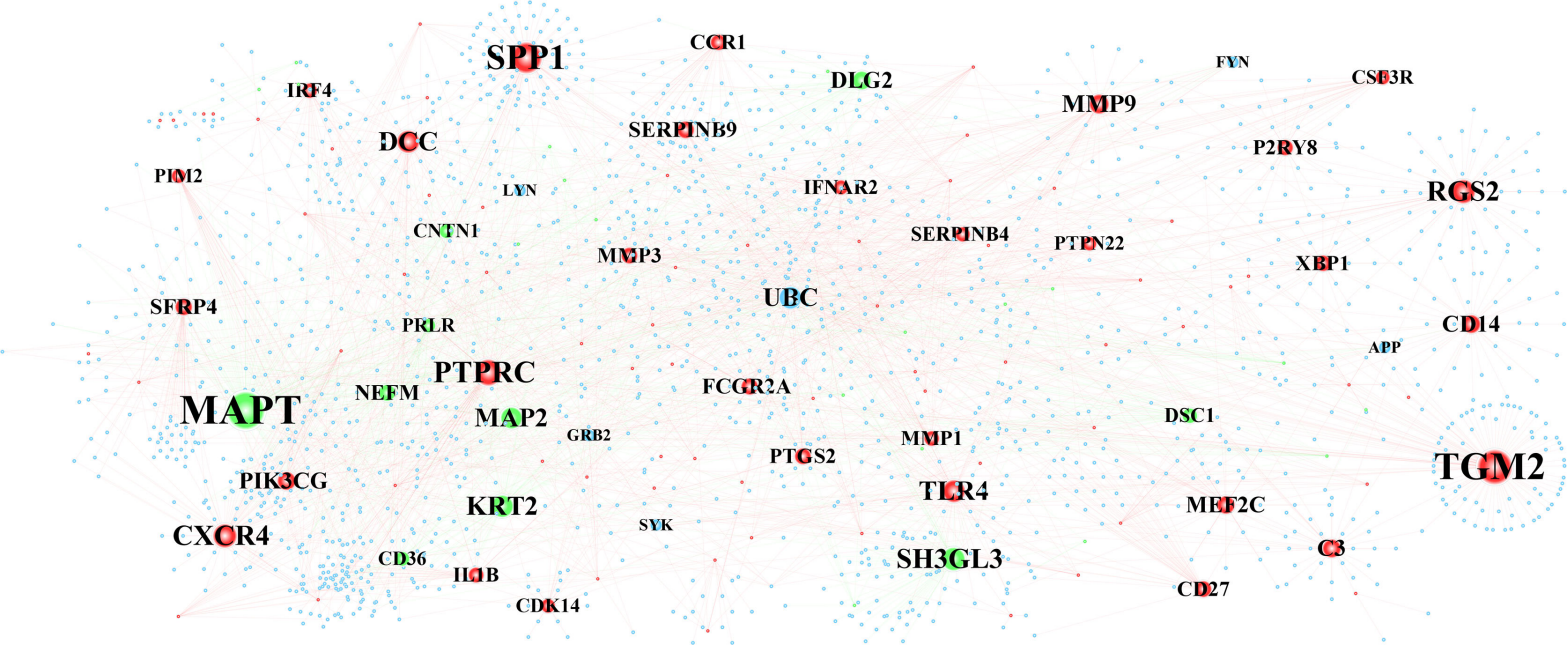
The protein–protein interaction network of periimplantitis.

The constructed PPI interwork of periimplantitis comprised 1,818 nodes and 2,517 edges (**Figure 4** and **Supplementary Figure 1A**). **Table 2** shows the top 30 nodes based on analysis of the network topological characteristics of PPI network. According to the topological characteristics, MAPT, TGM2, and SPP1 had the highest degree in the biological network, which may affect the development of periimplantitis.

**Table 2:** The topological characteristic of top 30 nodes in the PPI network for periimplantitis.

Gene	Label	Degree	Average shortest path length	Betweenness centrality	Closeness centrality	Topological coefficient
*MAPT*	DEG_down	125	2.987764	0.14551	0.334698	0.021494
*TGM2*	DEG_up	111	3.066741	0.117794	0.326079	0.014742
*SPP1*	DEG_up	96	3.604004	0.098392	0.277469	0.01648
*PTPRC*	DEG_up	76	2.967186	0.102146	0.33702	0.023215
*RGS2*	DEG_up	66	3.249166	0.069715	0.307771	0.021613
*CXCR4*	DEG_up	65	3.069522	0.071465	0.325784	0.021953
*SH3GL3*	DEG_down	63	3.361513	0.063809	0.297485	0.021645
*TLR4*	DEG_up	60	3.186318	0.065418	0.313842	0.022556
*DCC*	DEG_up	60	3.296997	0.077936	0.303306	0.020464
*KRT2*	DEG_down	59	3.24416	0.050792	0.308246	0.035569
*MAP2*	DEG_down	56	3.214683	0.050585	0.311073	0.03229
*UBC*	DEG_up	55	2.562291	0.426946	0.390276	0.023558
*MMP9*	DEG_up	48	3.914905	0.033259	0.255434	0.037429
*DLG2*	DEG_down	43	4.176863	0.034852	0.239414	0.031712
*PIK3CG*	DEG_up	43	3.504449	0.037451	0.285352	0.031464
*CD14*	DEG_up	43	3.768632	0.039586	0.265348	0.025859
*C3*	DEG_up	43	3.239711	0.041174	0.30867	0.026112
*MEF2C*	DEG_up	41	3.229143	0.040502	0.30968	0.035192
*SERPINB9*	DEG_up	38	3.319244	0.033189	0.301273	0.037007
*SFRP4*	DEG_up	37	4.374305	0.034312	0.228608	0.039501
*FCGR2A*	DEG_up	36	3.300334	0.038984	0.303	0.039652
*NEFM*	DEG_down	35	3.367631	0.030785	0.296945	0.039909
*PTGS2*	DEG_up	34	3.29366	0.027667	0.303614	0.045654
*MMP3*	DEG_up	34	3.921023	0.022136	0.255035	0.036284
*CD27*	DEG_up	34	4.190211	0.029729	0.238651	0.035294
*XBP1*	DEG_up	33	3.453838	0.036381	0.289533	0.034848
*P2RY8*	DEG_up	32	3.468854	0.033473	0.28828	0.032346
*CCR1*	DEG_up	32	4.384872	0.023611	0.228057	0.051339
*IL1B*	DEG_up	30	3.829811	0.021679	0.26111	0.048148
*MMP1*	DEG_up	29	3.299221	0.03134	0.303102	0.04902

PPI, protein–protein interaction.

### Transcription Factor–Gene Regulation Network

**Figure 5:**
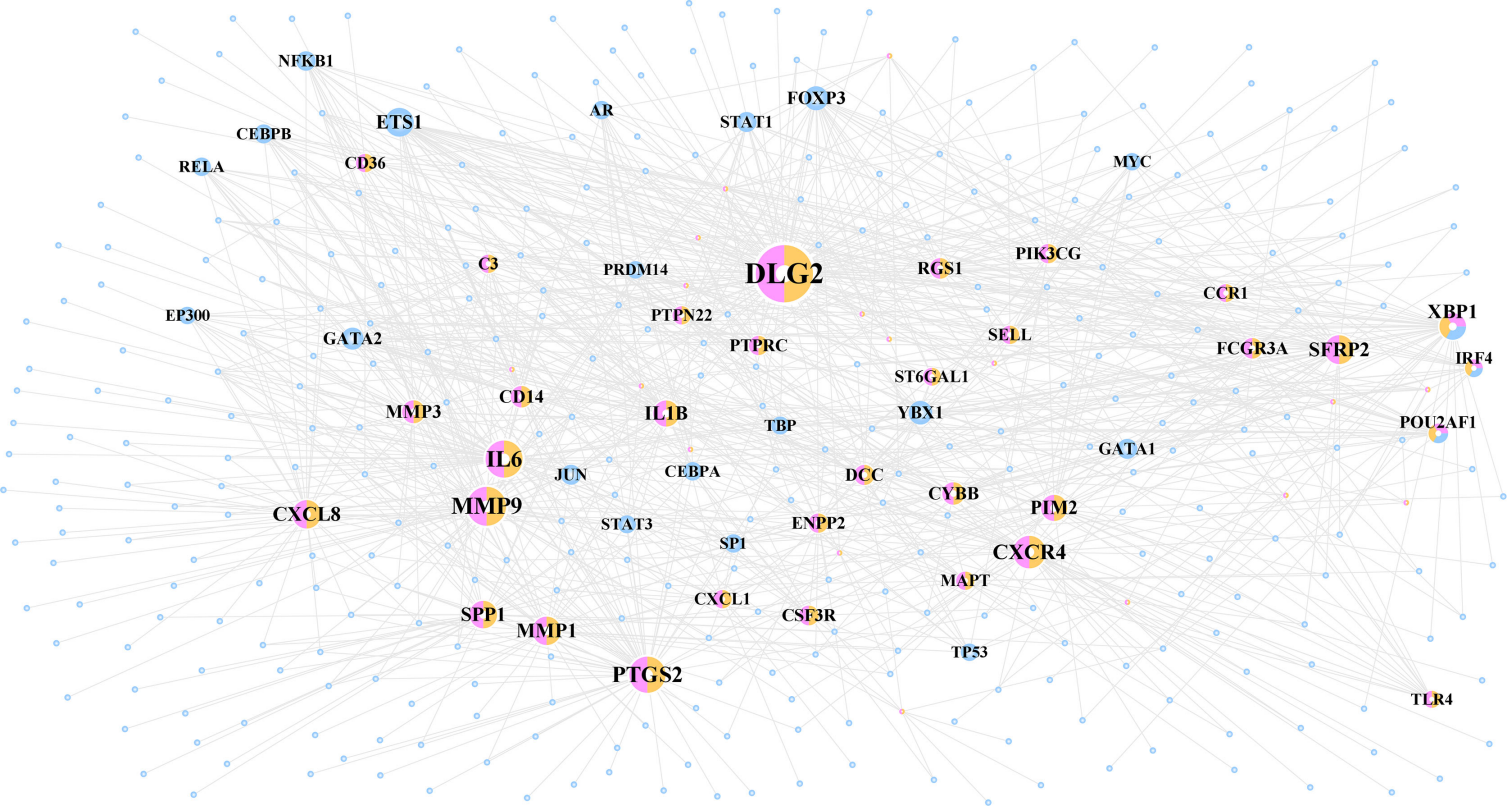
The TF–target network of periimplantitis. TF, transcription factor.

A total of 1,067 TF–target interactions were extracted, and the TF–target network was constructed as shown in **Figure 5** and **Supplementary Figure 1B**. The potential cross-talk genes with the highest degree were *DLG2*, *MMP9*, and *IL6* and therefore potentially played an important role in the TF–target network (**Table 3**).

**Table 3:** The topological characteristic of top 30 nodes in the TF–target network.

Node	Label	Degree	Average shortest path length	Betweenness centrality	Closeness centrality	Topological coefficient
DLG2	RA&DEG	101	2.291339	0.26366	0.436426	0.054709
MMP9	RA&DEG	61	2.566929	0.114349	0.389571	0.100499
IL6	RA&DEG	57	2.514436	0.107755	0.397704	0.084795
PTGS2	RA&DEG	55	2.606299	0.118554	0.383686	0.10303
CXCR4	RA&DEG	47	2.629921	0.09771	0.38024	0.099734
SFRP2	RA&DEG	39	2.669291	0.05225	0.374631	0.08637
MMP1	RA&DEG	38	2.708661	0.071448	0.369186	0.118115
CXCL8	RA&DEG	38	2.711286	0.063668	0.368829	0.07807
SPP1	RA&DEG	36	2.755906	0.07331	0.362857	0.10582
XBP1	RA&DEG&TF	34	2.309711	0.071833	0.432955	0.060406
PIM2	RA&DEG	33	2.761155	0.040276	0.362167	0.119146
IL1B	RA&DEG	33	2.740157	0.033118	0.364943	0.13447
ETS1	NA&TF	32	2.044619	0.111787	0.489089	0.080329
MMP3	RA&DEG	26	2.790026	0.023866	0.35842	0.161538
CYBB	RA&DEG	25	2.716535	0.031566	0.368116	0.116774
CD14	RA&DEG	23	2.755906	0.034871	0.362857	0.146739
FOXP3	NA&TF	23	2.498688	0.030215	0.40021	0.094879
YBX1	NA&TF	22	2.396325	0.041313	0.417306	0.093366
RGS1	RA&DEG	21	2.750656	0.020727	0.36355	0.093933
FCGR3A	RA&DEG	21	2.832021	0.027357	0.353105	0.180736
DCC	RA&DEG	20	2.88189	0.011931	0.346995	0.139024
POU2AF1	RA&DEG&TF	19	2.795276	0.014623	0.357746	0.143797
CSF3R	RA&DEG	19	2.845144	0.014582	0.351476	0.163743
GATA2	NA&TF	19	2.574803	0.022103	0.388379	0.102953
PTPRC	RA&DEG	18	2.805774	0.013921	0.356408	0.183951
PIK3CG	RA&DEG	18	2.821522	0.027588	0.354419	0.140152
ENPP2	RA&DEG	18	2.902887	0.013126	0.344485	0.172087
SELL	RA&DEG	16	2.826772	0.010231	0.35376	0.175
PTPN22	RA&DEG	16	2.821522	0.008702	0.354419	0.190341
MAPT	RA&DEG	16	2.908136	0.013747	0.343863	0.157095

TF, transcription factor.

### Prediction of Risk Cross-Talk Genes

**Figure 6:**
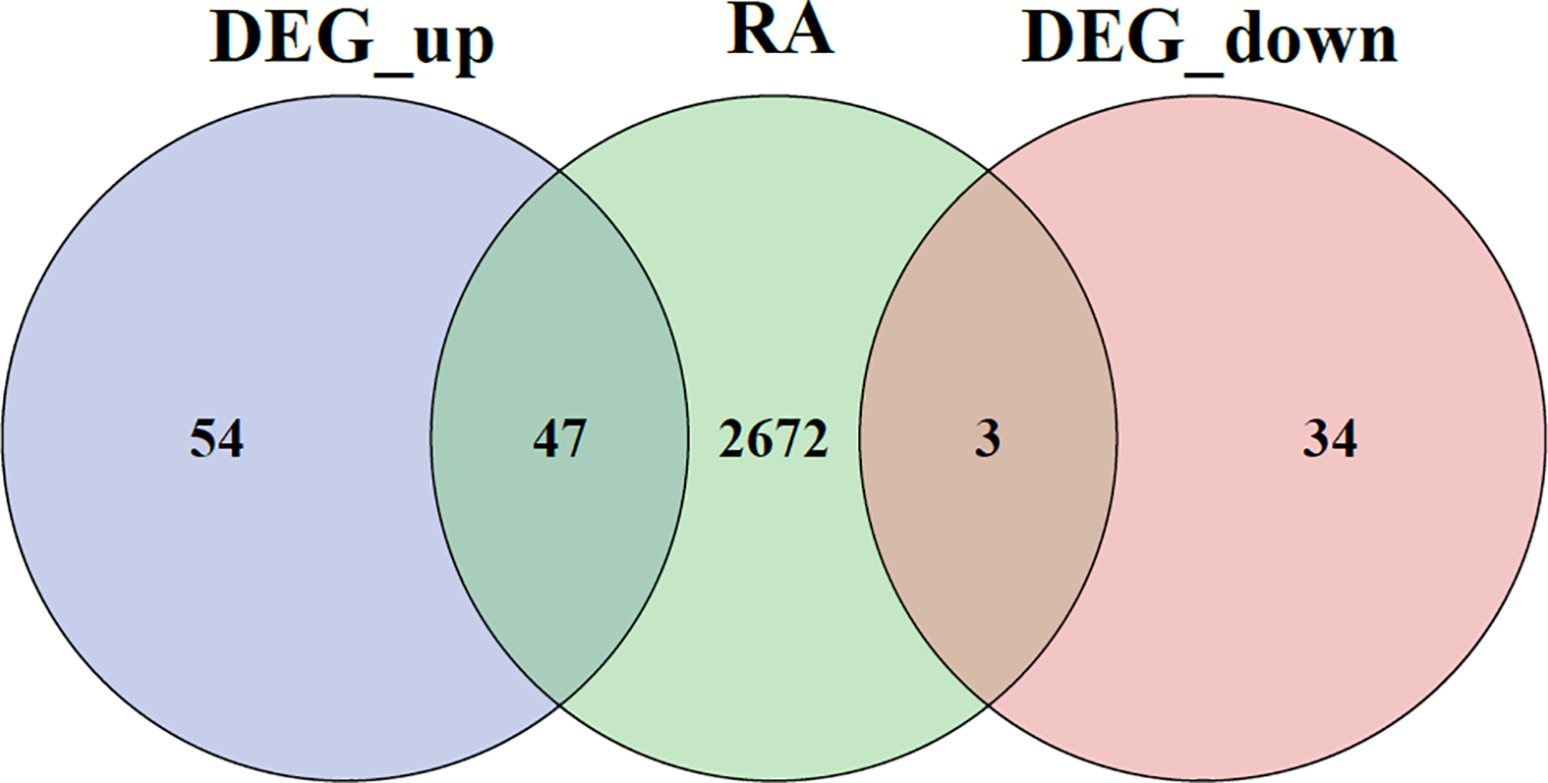
Venn diagram of DEGs. DEGs, differentially expressed genes.

A Venn diagram for DEGs is given in **Figure 6**.

**Figure 7:**
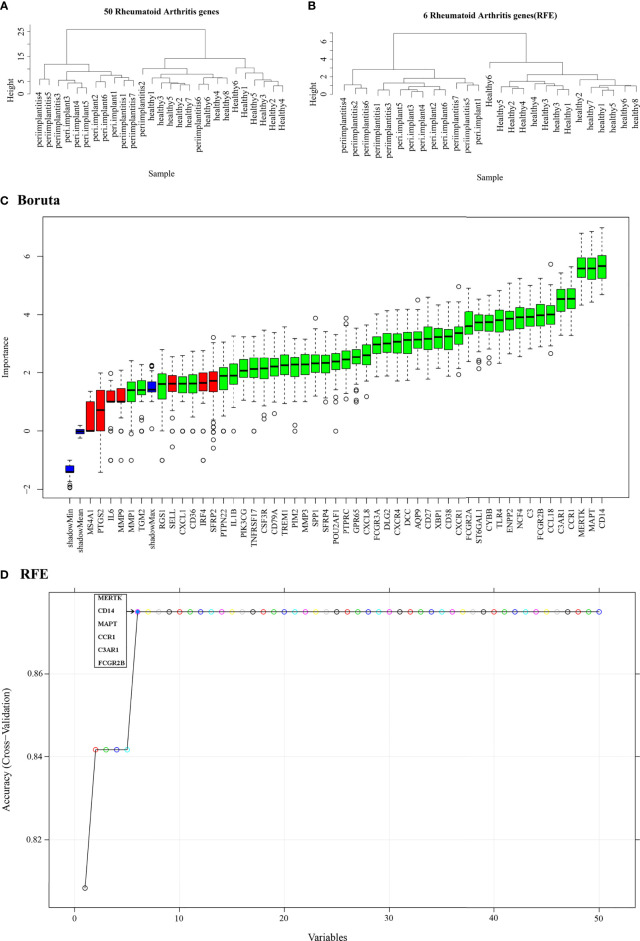
Feature selection. **(A)** Hierarchical clustering of 50 potential cross-talk genes in the merged data. **(B)** Hierarchical clustering of six feature genes that were selected by RFE algorithm in the merged data. **(C)** Cross-talk genes selected by using Boruta algorithm. **(D)** Six feature genes selected by using RFE algorithm. The abscissa of the figure is the variable of the number of genes, and the ordinate is the exact value of the whole dataset measured under this variable. The results show that when the minimum variable is 6, the score is high, which means that six features could map the entire dataset. RFE, Recursive Feature Elimination.

The normalized data were clustered, and the results showed that most of samples were clustered in the corrected groups (**Figure 7A**). RFE (**Figure 7D**) and Boruta (**Figure 7C**) selected the features. The results showed that there are more features with Boruta, and the RFE method selected six features (MERTK, CD14, MAPT, CCR1, C3AR1, and FCGR2B), which had the highest prediction. Therefore, the features of RFE were selected for subsequent analysis. The gene expression profile of the six feature genes from the merged data was extracted, and then sample hierarchical clustering was performed, which showed that the disease and the control groups could be clustered into two groups (**Figure 7B**). Therefore, the SVM model was built with these six genes.

According to the python scikit-learn package, the best hyperparameters of the SVM model(s) were obtained by using fivefold CV. The predicted sample type is periimplantitis group when the evaluation score was less than 0; otherwise, the predicted sample type belongs to the healthy control group. The results showed that the predicted sample types were consistent with the actual sample types (**Table 4**).

**Table 4:** Test sets for GSE33774 and GSE106090.

Sample types	SVM prediction types	SVM predicts scores	Accuracy
Healthy	Healthy	0.61043069	100%
Healthy	Healthy	0.70950425	
Healthy	Healthy	1.576055008	
Healthy	Healthy	0.381737285	
Periimplantitis	Periimplantitis	−0.172103955	
Periimplantitis	Periimplantitis	−1.026444558	
Healthy	Healthy	0.776789337	
Healthy	Healthy	0.511001026	
Periimplantitis	Periimplantitis	−0.793783932	

SVM, support vector machine.

### Support Vector Machine Modeling Using Feature Selection Cross-Talk Genes

To further verify the prediction of the merged model, the GSE33774 and GSE106090 were subsequently analyzed separately. According to the built prediction model, the results showed that the prediction of the merged model was done well for GSE33774 and GSE106090 separately (**Supplementary Tables 2, 3**).

### Relationship Prediction Between Periimplantitis and Rheumatoid Arthritis

A list of DEGs for RA is given in **Supplementary Table 4**. To further predict whether there is a cross-talk between periimplantitis and RA, the gene expression profile of six feature genes was extracted from the RA dataset GSE93272. Firstly, the gene expression value of GSE93272 was normalized and then imputed into the built model, which was established by the periimplantitis datasets. Fisher’s exact test showed that *p*-value was less than 0.05, confirming the prediction of six cross-talk genes for periimplantitis and RA (**Table 5**).

**Table 5:** The prediction of six feature genes in GSE93272.

GSE93272 (sample)	SVM predicts periimplantitis	SVM predicts non-periimplantitis	Fisher’s test (*p-*value)
Rheumatoid arthritis	110	122	0.00232
Healthy control	10	33	

SVM, support vector machine.

**Figure 8:**
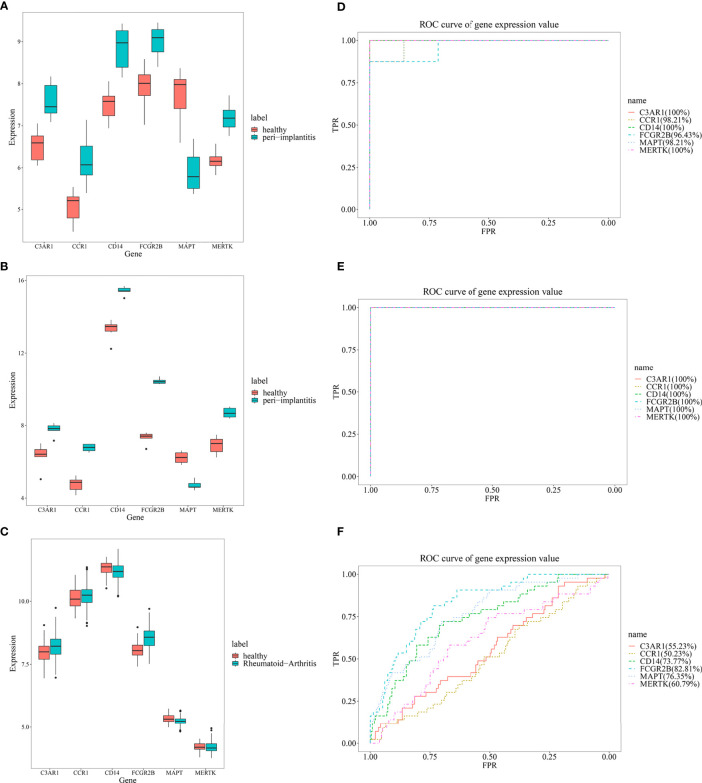
The expression levels of six feature genes in the **(A, B)** periimplantitis datasets (GSE33774 and GSE106090) and **(C)** RA datasets (GSE93272). The prediction of six feature genes in the periimplantitis datasets **(D, E)** (GSE33774 and GSE106090) and **(F)** RA dataset (GSE93272). RA, rheumatoid arthritis.

From the expression values of six feature genes in periimplantitis and RA, *CD14* and *FCGR2B* were most highly expressed in the periimplantitis and RA (**Figures 8A–C**). As shown in **Figures 8D–F**, the area under curve (AUC) values of CD14 and FCGR2B in the periimplantitis (GSE33774 and GSE106090) were over 95%. In the RA dataset (GSE93272), the AUC values of CD14 and FCGR2B were 73.77% and 82.81%, respectively.

### Pathway–Gene Functional Network

**Figure 9:**
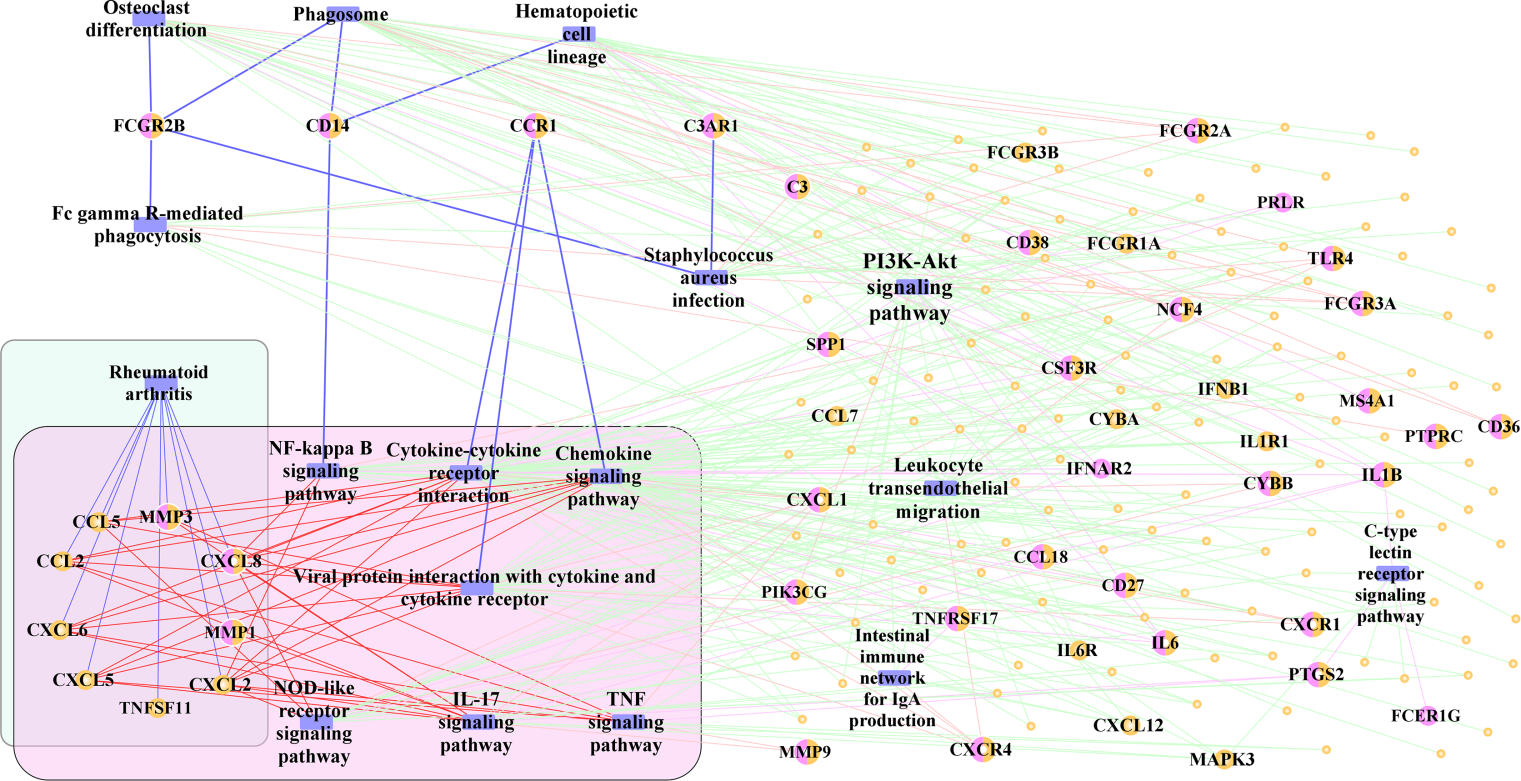
The activated pathway–gene network.

Finally, 17 significant pathways, which may play an important role in the development of periimplantitis, were selected. To identify the pathway cross-talk between periimplantitis and RA, the pathway–gene cross-talk network was constructed. This activated pathway–gene network contained 181 nodes and 360 edges (**Figure 9** and **Supplementary Figure 2A**). There were four feature genes (*CD14*, *CCR1*, *C3AR1*, and *FCGR2B*) in the activated pathway–gene network. For example, CD14 regulated the NF-kappa B signaling pathway, which would induce the dysregulation of CXCL8 and CXCL2. CXCL8 was differentially expressed in the periimplantitis and regulated the pathways of RA, too.

**Figure 10:**
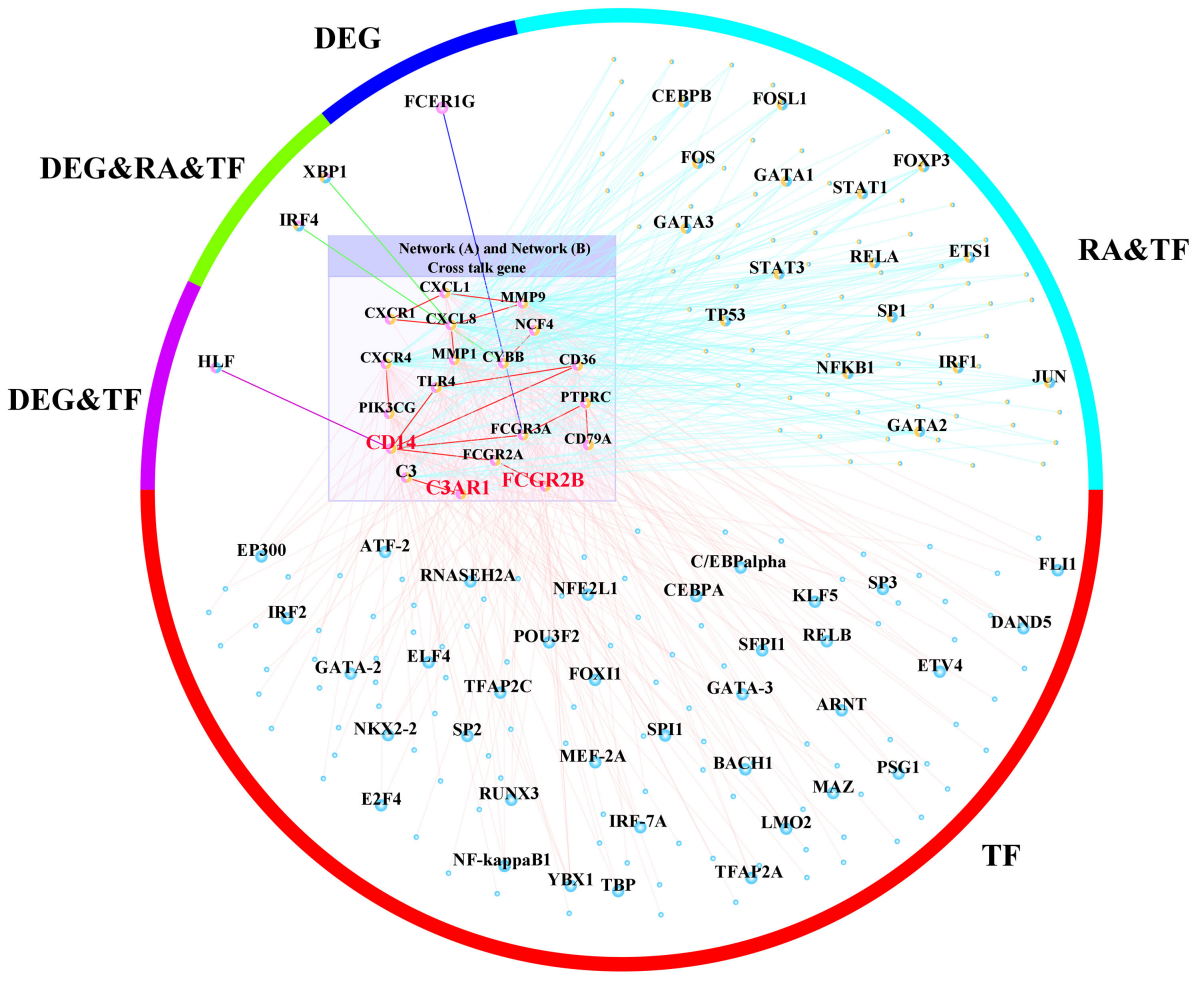
The activated TF–target network. TF, transcription factor.

To further identify the regulation of TF in the cross-talk pathways between periimplantitis and RA, the cross-talk genes from the activated pathways and TF from the TF–target relationships were extracted. Meanwhile, the PPI between cross-talk genes was acquired, and the activated TF–cross-talk gene network was built (**Figure 10** and **Supplementary Figure 2B**). Thereby, it was found that cross-talk genes were regulated by many TFs.

## Discussion

The main result of the current study was that the bioinformatics analysis was able to reveal cross-talk genes between periimplantitis and RA. Thereby, *FCGR2B* and *CD14* were found to be the most relevant genes, and several related pathways were identified. The potential relevance of these genes is seen based on their expression values in the disease samples and their related predictive effects. Because the ROC values of these two genes are higher than those of other genes, they were considered to have the highest correlation. Meanwhile, CD14 interacted with FCGR2B through FCGR2A, which means that CD14 would be potentially related to RA through interacting with other genes. Accordingly, this discussion primarily focuses on this relation, although this renders it somewhat speculative.

The datasets for periimplantitis samples originated from two clinical studies, which examined RNA expression profiles between periimplantitis and periodontitis (24, 25). As the overlap between periimplantitis and RA was not addressed in those previous studies, a comparison is difficult. Generally, periimplantitis is a complex inflammatory disease, of which etiology and pathogenesis are still not fully understood (11, 16). Primarily, a heterogenic biofilm of potentially pathogenic, opportunistic microorganisms appears to be essential for establishment of inflammation; however, genetic predisposition alongside with different immunological issues might be of high relevance in periimplantitis development and progression (16). Thereby, the microbiome of periimplantitis is complex; alongside with a heterogenic and various composition of microorganisms, prevalence of potentially periodontal pathogenic bacteria can be increased in inflamed periimplant tissues (26). Similarly, the microbiome has been evaluated as an important point to understand RA-related autoimmunity (27). Due to the heterogeneity of microorganisms and potentially related pathways, the significance of the current study’s findings with regard to the related microbiome cannot be estimated, yet. Another potentially related indicator from clinical perspective could be smoking, which was found to be related to both periimplantitis and RA (28, 29). Accordingly, a potential interlink between periimplantitis and RA could be conceivable based on such shared risk factors. Especially, smoking is known to affect DNA methylation of blood cells, potentially mediating disease progression and affecting immune cell function; in this respect, smoking also affects CD14+ monocytes (30). As CD14 was found to be one of the most important genes in the current study, the hypothesis of a relevance of shared risk factors like smoking could be of interest. Thus, further parameters like comorbidities, including diabetes mellitus, could be of relevance. It is documented that the presence of diabetes mellitus can increase the risk for periimplantitis as well as RA (31, 32). In context of oral inflammation (periodontitis), pro-inflammatory CD14+ monocytes were clearly increased in diabetic individuals (33). Although the consideration of risk factors (like smoking) and comorbidities (like diabetes mellitus) increases the complexity of the topic, this approach needs to be recognized in the interpretation of the current findings. Based on the current state of knowledge, the related pathways remain unclear and can be a focus of subsequent studies.

The understanding of an implant as foreign body, which causes a transient foreign body reaction in which the organism can hold a respective equilibrium or dysbalance, is of interest (14, 15). Thereby, histologically visible giant cells can occur on osseointegrated implants as a result of foreign body reaction, which are similar as giant cells in rheumatically inflamed cartilage tissue (14). Accordingly, it can be presumed that periimplantitis is a result of immune-osteolytic reactions, which is similar to an autoimmune reaction (14, 15). Therefore, a similarity or even relationship with RA appears a plausible approach, and this is supported by the presence of cross-talk genes and shared pathways as confirmed in the current study.

One of the two most relevant cross-talk genes revealed in the current study was CD14. CD14 is a differentiation antigen, enhancing innate immune response (34). A clinical study examining 180 patients with periimplantitis and 189 controls was able to show CD14-159 polymorphism to be associated with periimplantitis (35). Another clinical examination showed CD14-159 CC/CT genotype to decrease the risk of periimplantitis (36). Therefore, CD14 appears to be a potential candidate DEG in periimplantitis. Moreover, CD14 was also found to play a role in RA pathogenesis; CD14-positive monocytes from RA patients induct pro-inflammatory processes (37). It is suggested that CD14+ monocytes can differentiate to osteoclasts, leading to bone erosion of the synovial joint tissue in RA (38). Thereby, CD14+ monocytes are critical in osteoclast differentiation and thus bone resorption in RA-diseased individuals (39). Accordingly, activation of CD14 in monocytes could increase the risk of periimplant inflammation and bone loss and could therefore explain an interrelationship between RA and periimplantitis. Moreover, nuclear factor kappa B (NFkB) was found to be a shared pathway between RA and periimplantitis in the current analysis. This pathway is known to play an important role in RA pathogenesis (40). In periimplantitis, the NFkB ligand (RANKL) has been reported to be of importance (41). Interestingly, RANKL is increased by the action of lipopolysaccharides from *Porphyromonas gingivalis* (Pg) in the periimplant tissue (42). *Pg* is also discussed to be involved in the RA pathogenesis due to its ability of citrullination of antibodies and furthermore its influence on osteoclastogenesis and systemic inflammation (43). Altogether, CD14 appears to be a hint of an osteoimmunologic relationship or even similarity between periimplantitis and RA; it is known that immune-inflammatory-induced uncoupled bone remodeling is crucial in periimplant disease (44). A previous study based on RNA expression analysis confirmed an upregulated osteoclast differentiation in periimplantitis, which was higher compared with periodontitis (25). An increasing number of CD14+ monocytes in RA individuals results in increased transcription of IL-6 and IL-23 (45). Titan particles originating from dental implants were reported to lead to increased IL-6 expression by macrophages, resulting in a stimulation of osteoclasts and thus into bone resorption around dental implants (46). This supports the abovementioned theory of a foreign body reaction around dental implants, which seems similar as auto-inflammatory processes in rheumatic disease and potentially related to an altered expression of CD14.

The second most relevant cross-talk gene was *FCGR2B*, which is an inhibitory receptor with a central role in the regulation of autoantibody generation and thus autoimmunity (47). This gene has not been described in context of periimplantitis, but only related to periodontitis (48). For RA, FCGR2B can be dysregulated in active disease (49). Especially, the shared pathway of osteoclast differentiation might be of relevance in the interrelation between RA and periimplantitis in this context. An immune-inflammatory-induced osteoclast differentiation appears to be a major pathogenic aspect of periimplantitis, as already mentioned above (44). In RA pathogenesis, the stimulation of differentiation of bone-resorbing osteoclasts is an important process leading to bone erosion in inflamed joints (50). The increased tendency to increased osteoclast activity could explain the shift from a foreign body equilibrium in the periimplant tissue into a progredient, dysbalanced inflammation leading to bone damage. Furthermore, MMP3 and MMP1 were found to be of potential relevance in the network within the current analysis. These collagenases were reported to be of relevance in both RA and periimplantitis (51, 52). From the clinical perspective, this might be a further point of interrelationship between the two diseases, because a dysbalance of collagenase activity in RA individuals with periodontal inflammation has already been observed (8). Altogether, the current study revealed two major cross-talk genes and several shared pathways, which underline the similarity and a potential relationship between RA and periimplantitis. It is conceivable that RA patients would develop earlier and more severe/progredient bone damage at dental implants caused by an increased inflammatory potential and osteoclast activity. Regardless of the plausibility of such a potential interlink, clinical data in this respect are very scarce up until now; only one clinical study with a low sample size of 34 patients is available, showing no hints for more severe periimplantitis in RA individuals (19). Altogether, the clinical body of evidence is low, the reason that systematic reviews on systemic risk factors for periimplantitis concluded a necessity for more research in the field (17, 18). Based on available clinical data, no statement on the practical significance of the prediction results of this current study is possible, underlining the need for validation and further research in the field.

### Strengths and Limitations

This is the first analysis of cross-talk mechanisms between periimplantitis and RA on the transcriptomic level. Bioinformatics analysis has been applied for several other diseases and conditions, such as periodontitis and Alzheimer’s disease, periodontitis and oral cancer, or between periimplantitis and type 2 diabetes mellitus (20, 21, 53). Therefore, this method appears reasonable for the current study aim, i.e., detecting the cross-talk between periimplantitis and RA to reveal whether there is a similarity regarding auto-inflammation between the two diseases. It must thereby be recognized that the associated patterns shown in this manuscript are correlations at the transcriptomic level and provide no information on shared genetic mechanisms. The current analysis does not provide a causal evidence at all. The analyses were comprehensive and included PPI network, TF, and pathway analyses. The major limitation is the low sample size of datasets and the low availability of data for periimplantitis. Since only 27 samples were obtained by combining the two datasets after batch correction, in order to generate more training data, the proportion of training set and test set was selected as 70/30. As presented in **Table 4**, the discovery model can predict the results of the test set well. Additionally, expression values of characteristic genes in the extracted GSE33774 and GSE106090 datasets were predicted before correction, and the results were also appropriate (**Supplementary Tables 2** and **3**). This indicates that although the sample size used in the establishment of SVM is small, it has reached the demand of predicting whether the unknown samples have periimplantitis (or are at high risk for having periimplantitis) through six characteristic genes. To increase the value of conclusions, samples were both merged and analyzed separately to verify that the results are as good as possible. It must be considered that primarily two genes the focus of the discussion here. When selecting few genes from an entire set, further support of the results, e.g., an expression quantitative trait locus (eQTL) analysis, would strengthen the significance of findings. However, the current study did not analyze single-nucleotide polymorphisms (SNP), and mutations of SNP cannot be assessed from the available datasets. If the 1000 Genomes Project would be used, the expression level of corresponding genes would not be accessible, and it would be unclear, if samples were affected by either periimplantitis or RA. Regarding the analyzed samples, the missing analysis of sample information for RA and several periimplantitis samples, e.g., age, gender, comorbidities, lifestyle, and analytic procedures, must be seen as a limitation. Therefore, the criteria of data selection and comparison are simplified in such an analysis, which is an important limitation of the interpretability of the findings and the ability to draw robust conclusions. Since finally only three downregulated DEGs were obtained, they cannot be used for functional enrichment analysis. Therefore, the enriched biological function of all DEGs (up- and downregulated) was evaluated. The results obtained by the applied enrichment method show that the regulation pattern of genes is not related with the activation or inactivation of functions of genes. It was only analyzed, which functions were dysregulated by these DEGs, from the perspective of statistics and methodology. The specific regulation pattern (up- or downregulation) needs to be verified by future experiments. Although the revealed results appear reasonable and plausible, the interpretation is mostly speculative. To confirm the findings, studies need to be performed, which examine the cellular mechanisms *in vitro* and/or in a clinical study setting. Accordingly, the current study’s results can serve as a theoretical basis for future research in the field.

## Conclusion

This current bioinformatics study revealed *FCGR2B* and *CD14* as the most relevant potential cross-talk genes between RA and periimplantitis on the transcriptomic level. These were related to potentially relevant pathways, including NFkB and osteoclast differentiation. These findings suggest a similarity and a potential relationship between RA and periimplantitis and can serve as a theoretical basis for future research.

## Data Availability Statement

The original contributions presented in the study are included in the article/**Supplementary Material**. Further inquiries can be directed to the corresponding author.

## Author Contributions

SL, CZ, YX, and ZQ designed the study, performed data analysis, prepared the figures and tables, wrote the manuscript and approved the final draft. YW, LL, GP, and DZ participated in data interpretation and analysis, were involved in proofreading and deep editing and approved the final manuscript. GS and ZQ devised the main conceptual idea and supervised the project, performed proofreading and deep editing of the manuscript and approved the final draft. All authors contributed to the article and approved the submitted version.

## Funding

This study was supported by the National Natural Science Foundation of China (Grant No.: 81902767) and Natural Science Foundation of Guangdong Province (Grant No.: 2020A1515011352).

## Conflict of Interest

The authors declare that the research was conducted in the absence of any commercial or financial relationships that could be construed as a potential conflict of interest.

## Publisher’s Note

All claims expressed in this article are solely those of the authors and do not necessarily represent those of their affiliated organizations, or those of the publisher, the editors and the reviewers. Any product that may be evaluated in this article, or claim that may be made by its manufacturer, is not guaranteed or endorsed by the publisher.
