# Supplementary figures and images for: Similarity and Potential Relation Between Periimplantitis and Rheumatoid Arthritis on Transcriptomic Level: Results of a Bioinformatics Study
Source: Front Immunol. 2021 Nov 9;12:702661. doi: 10.3389/fimmu.2021.702661 (PMC8630748; doi:10.3389/fimmu.2021.702661)

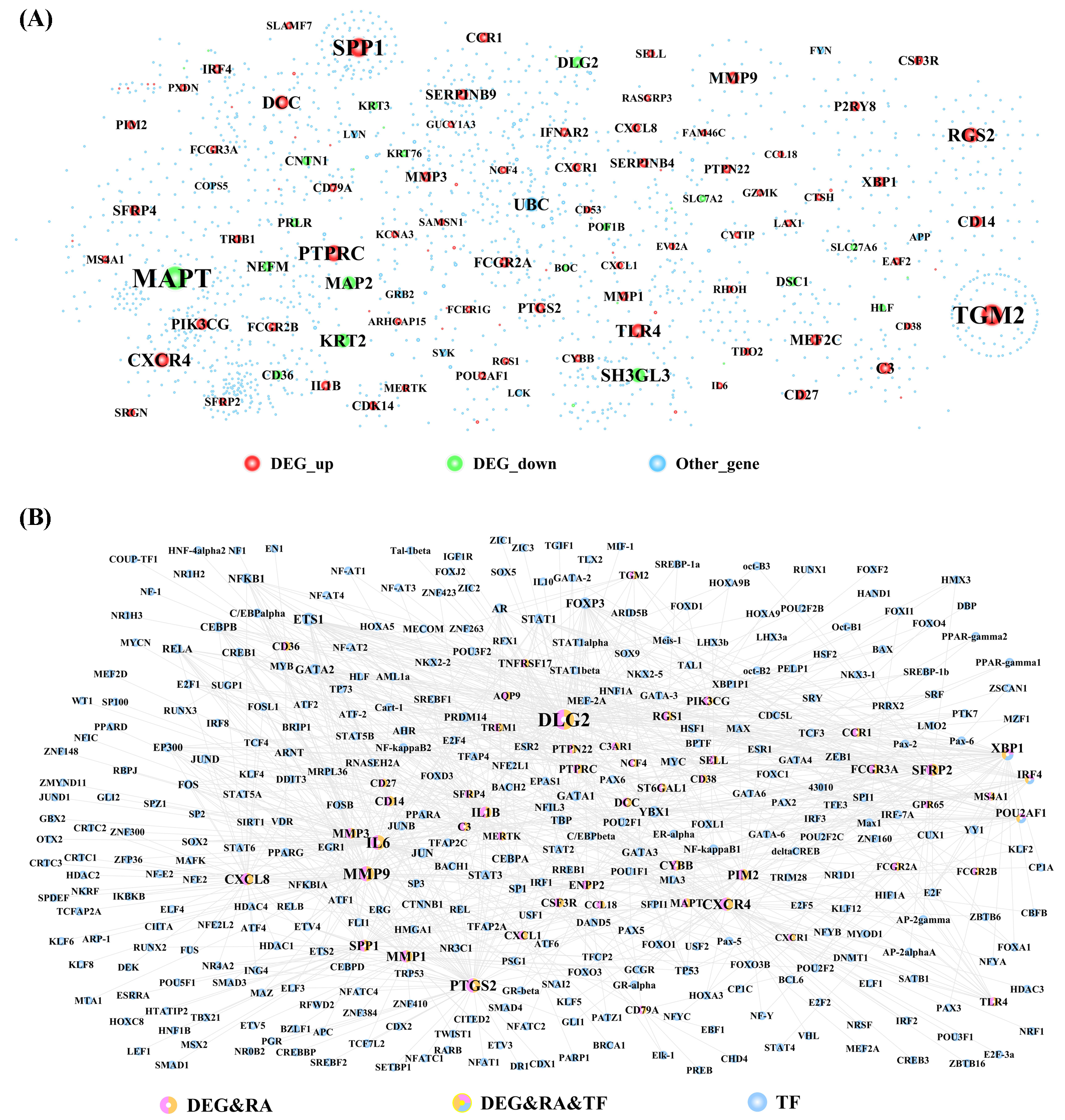

Supplement: Supplementary file 1 [file Image_1.tif]

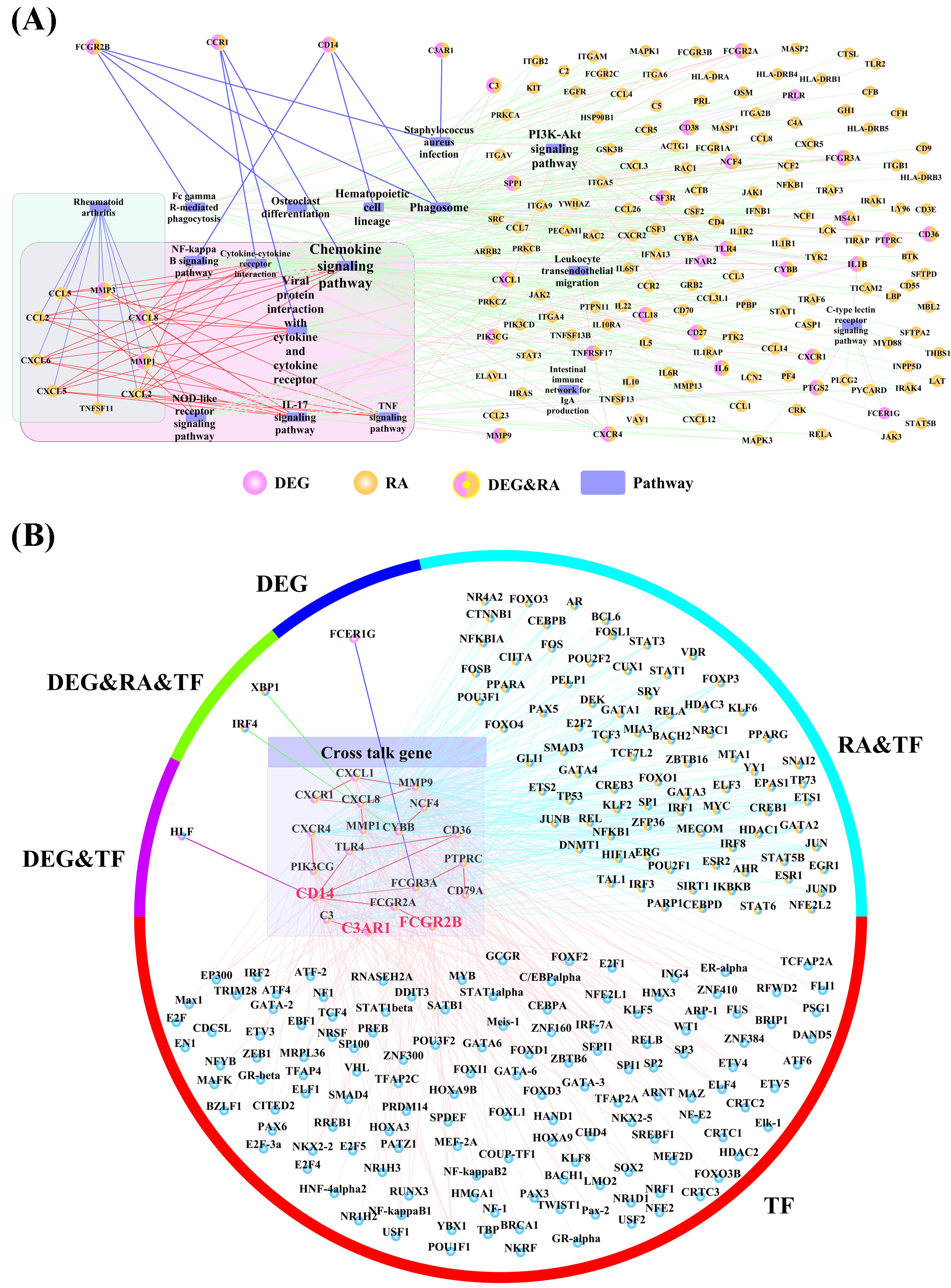

Supplement: Supplementary file 2 [file Image_2.tif]
