# Supplementary material for: Similarity and Potential Relation Between Periimplantitis and Rheumatoid Arthritis on Transcriptomic Level: Results of a Bioinformatics Study
Source: Front Immunol. 2021 Nov 9;12:702661. doi: 10.3389/fimmu.2021.702661 (PMC8630748; doi:10.3389/fimmu.2021.702661)
Supplement: Supplementary file 3 [file Table_1.docx]

**Supplementary files**

**Supplementary table 1:** Sample characteristics of periimplantitis and control samples.

| **sample** | **Age, gender** | **Cell type** | **Amplification** | **Analysis** | **Processing** |
| --- | --- | --- | --- | --- | --- |
| **Periimplantitis** | | | | | |
| GSM2829419 | 42y,male | Inflamed periimplant tissue | Quick Amp Labeling Kit, One-Color (part number 5190-0442, Agilent, CA, USA), RNeasy Mini Kit (Qiagen, CA, USA). NanoDrop ND-1000 | Agilent Microarray Scanner (part number G2565BA, Agilent, CA, USA) | Agilent Feature Extraction software (version 11.0.1.1) GeneSpring GX v12.1 software package (Agilent Technologies) |
| GSM2829420 | 63y, female | Inflamed periimplant tissue | Quick Amp Labeling Kit, One-Color (part number 5190-0442, Agilent, CA, USA), RNeasy Mini Kit (Qiagen, CA, USA). NanoDrop ND-1000 | Agilent Microarray Scanner (part number G2565BA, Agilent, CA, USA) | Agilent Feature Extraction software (version 11.0.1.1) GeneSpring GX v12.1 software package (Agilent Technologies) |
| GSM2829421 | 47y, female | Inflamed periimplant tissue | Quick Amp Labeling Kit, One-Color (part number 5190-0442, Agilent, CA, USA), RNeasy Mini Kit (Qiagen, CA, USA). NanoDrop ND-1000 | Agilent Microarray Scanner (part number G2565BA, Agilent, CA, USA) | Agilent Feature Extraction software (version 11.0.1.1) GeneSpring GX v12.1 software package (Agilent Technologies) |
| GSM2829422 | 46y, male | Inflamed periimplant tissue | Quick Amp Labeling Kit, One-Color (part number 5190-0442, Agilent, CA, USA), RNeasy Mini Kit (Qiagen, CA, USA). NanoDrop ND-1000 | Agilent Microarray Scanner (part number G2565BA, Agilent, CA, USA) | Agilent Feature Extraction software (version 11.0.1.1) GeneSpring GX v12.1 software package (Agilent Technologies) |
| GSM2829423 | 76y, male | Inflamed periimplant tissue | Quick Amp Labeling Kit, One-Color (part number 5190-0442, Agilent, CA, USA), RNeasy Mini Kit (Qiagen, CA, USA). NanoDrop ND-1000 | Agilent Microarray Scanner (part number G2565BA, Agilent, CA, USA) | Agilent Feature Extraction software (version 11.0.1.1) GeneSpring GX v12.1 software package (Agilent Technologies) |
| GSM2829424 | 60y, male | Inflamed periimplant tissue | Quick Amp Labeling Kit, One-Color (part number 5190-0442, Agilent, CA, USA), RNeasy Mini Kit (Qiagen, CA, USA). NanoDrop ND-1000 | Agilent Microarray Scanner (part number G2565BA, Agilent, CA, USA) | Agilent Feature Extraction software (version 11.0.1.1) GeneSpring GX v12.1 software package (Agilent Technologies) |
| GSM835242 | 51y, female | primary tissue, biopsy | RNeasy (Qiagen) | Affymetrix GeneChip Scanner 3000 | Not applicable |
| GSM835243 | 57y, female | primary tissue, biopsy | RNeasy (Qiagen) | Affymetrix GeneChip Scanner 3000 | Not applicable |
| GSM835244 | 54y, female | primary tissue, biopsy | RNeasy (Qiagen) | Affymetrix GeneChip Scanner 3000 | Not applicable |
| GSM835245 | 63y, female | primary tissue, biopsy | RNeasy (Qiagen) | Affymetrix GeneChip Scanner 3000 | Not applicable |
| GSM835246 | 38y, female | primary tissue, biopsy | RNeasy (Qiagen) | Affymetrix GeneChip Scanner 3000 | Not applicable |
| GSM835247 | 71y, female | primary tissue, biopsy | RNeasy (Qiagen) | Affymetrix GeneChip Scanner 3000 | Not applicable |
| GSM835248 | 64y, female | primary tissue, biopsy | RNeasy (Qiagen) | Affymetrix GeneChip Scanner 3000 | Not applicable |
| **Healthy control** | | | | | |
| GSM2829431 | 31y, male | Healthy periodontal tissue | Quick Amp Labeling Kit, One-Color (part number 5190-0442, Agilent, CA, USA), RNeasy Mini Kit (Qiagen, CA, USA). NanoDrop ND-1000 | Agilent Microarray Scanner (part number G2565BA, Agilent, CA, USA) | Agilent Feature Extraction software (version 11.0.1.1) GeneSpring GX v12.1 software package (Agilent Technologies) |
| GSM2829432 | 33y, male | Healthy periodontal tissue | Quick Amp Labeling Kit, One-Color (part number 5190-0442, Agilent, CA, USA), RNeasy Mini Kit (Qiagen, CA, USA). NanoDrop ND-1000 | Agilent Microarray Scanner (part number G2565BA, Agilent, CA, USA) | Agilent Feature Extraction software (version 11.0.1.1) GeneSpring GX v12.1 software package (Agilent Technologies) |
| GSM2829433 | 46y, male | Healthy periodontal tissue | Quick Amp Labeling Kit, One-Color (part number 5190-0442, Agilent, CA, USA), RNeasy Mini Kit (Qiagen, CA, USA). NanoDrop ND-1000 | Agilent Microarray Scanner (part number G2565BA, Agilent, CA, USA) | Agilent Feature Extraction software (version 11.0.1.1) GeneSpring GX v12.1 software package (Agilent Technologies) |
| GSM2829434 | 28y, female | Healthy periodontal tissue | Quick Amp Labeling Kit, One-Color (part number 5190-0442, Agilent, CA, USA), RNeasy Mini Kit (Qiagen, CA, USA). NanoDrop ND-1000 | Agilent Microarray Scanner (part number G2565BA, Agilent, CA, USA) | Agilent Feature Extraction software (version 11.0.1.1) GeneSpring GX v12.1 software package (Agilent Technologies) |
| GSM2829435 | 38y, female | Healthy periodontal tissue | Quick Amp Labeling Kit, One-Color (part number 5190-0442, Agilent, CA, USA), RNeasy Mini Kit (Qiagen, CA, USA). NanoDrop ND-1000 | Agilent Microarray Scanner (part number G2565BA, Agilent, CA, USA) | Agilent Feature Extraction software (version 11.0.1.1) GeneSpring GX v12.1 software package (Agilent Technologies) |
| GSM2829436 | 23y, male | Healthy periodontal tissue | Quick Amp Labeling Kit, One-Color (part number 5190-0442, Agilent, CA, USA), RNeasy Mini Kit (Qiagen, CA, USA). NanoDrop ND-1000 | Agilent Microarray Scanner (part number G2565BA, Agilent, CA, USA) | Agilent Feature Extraction software (version 11.0.1.1) GeneSpring GX v12.1 software package (Agilent Technologies) |
| GSM835234 | 64y, male | primary tissue, biopsy | RNeasy (Qiagen) | Affymetrix GeneChip Scanner 3000 | Not applicable |
| GSM835235 | 42y, male | primary tissue, biopsy | RNeasy (Qiagen) | Affymetrix GeneChip Scanner 3000 | Not applicable |
| GSM835236 | 33y, male | primary tissue, biopsy | RNeasy (Qiagen) | Affymetrix GeneChip Scanner 3000 | Not applicable |
| GSM835237 | 78y, male | primary tissue, biopsy | RNeasy (Qiagen) | Affymetrix GeneChip Scanner 3000 | Not applicable |
| GSM835238 | 31y, male | primary tissue, biopsy | RNeasy (Qiagen) | Affymetrix GeneChip Scanner 3000 | Not applicable |
| GSM835239 | 29y, male | primary tissue, biopsy | RNeasy (Qiagen) | Affymetrix GeneChip Scanner 3000 | Not applicable |
| GSM835240 | 15y, female | primary tissue, biopsy | RNeasy (Qiagen) | Affymetrix GeneChip Scanner 3000 | Not applicable |
| GSM835241 | 14y, female | primary tissue, biopsy | RNeasy (Qiagen) | Affymetrix GeneChip Scanner 3000 | Not applicable |

**Supplementary table 2:** the prediction of 6 feature genes in GSE33774

| Sample types | SVM prediction types | SVM predicts scores | Accuracy |
| --- | --- | --- | --- |
| healthy | healthy | 0.576811345 | 93.3% |
| healthy | healthy | 0.761989893 |  |
| healthy | healthy | 0.738645274 |  |
| healthy | healthy | 0.60207661 |  |
| periimplantitis | periimplantitis | -1.296027179 |  |
| healthy | healthy | 0.542289721 |  |
| periimplantitis | periimplantitis | -0.069803436 |  |
| periimplantitis | periimplantitis | -1.345204005 |  |
| healthy | healthy | 0.907156812 |  |
| periimplantitis | periimplantitis | -0.389834745 |  |
| healthy | healthy | 1.042720056 |  |
| healthy | healthy | 0.754890829 |  |
| periimplantitis | periimplantitis | -0.752688722 |  |
| periimplantitis | healthy | 0.108990816 |  |
| periimplantitis | periimplantitis | -0.968064029 |  |

**Supplementary table 3:** the prediction of 6 feature genes in GSE106090

| Sample types | SVM prediction types | SVM predicts scores | Accuracy |
| --- | --- | --- | --- |
| periimplantitis | periimplantitis | -0.897104158 | 100% |
| periimplantitis | periimplantitis | -0.830668339 |  |
| periimplantitis | periimplantitis | -0.823269174 |  |
| periimplantitis | periimplantitis | -0.840090977 |  |
| healthy | healthy | 0.827264336 |  |
| periimplantitis | periimplantitis | -0.501118284 |  |
| healthy | healthy | 0.713390362 |  |
| periimplantitis | periimplantitis | -0.78141275 |  |
| healthy | healthy | 0.8557551 |  |
| healthy | healthy | 0.635474936 |  |
| healthy | healthy | 0.899651485 |  |
| healthy | healthy | 1.444417148 |  |

**Supplementary Table 4:** List of deregulated genes in RA(**Rheumatoid Arthritis**).**Disease_id :C0003873**

| **PD&RA(Gene)** | **Gene_id** | **logFC(GSE33774)** | **Pvalue(GSE33774)** | **logFC(GSE106090)** | **Pvalue(GSE106090)** | **Regulate** |
| --- | --- | --- | --- | --- | --- | --- |
| **AQP9** | **366** | **1.526774** | **0.005378** | **1.207112** | **9.83E-07** | **DEG_up** |
| **C3** | **718** | **1.689487** | **8.54E-05** | **1.952227** | **9.05E-08** | **DEG_up** |
| **C3AR1** | **719** | **1.079489** | **2.47E-05** | **1.462157** | **0.000354** | **DEG_up** |
| **CCL18** | **6362** | **2.242684** | **0.0019** | **4.687278** | **6.12E-07** | **DEG_up** |
| **CCR1** | **1230** | **1.107229** | **0.000196** | **2.017876** | **1.64E-07** | **DEG_up** |
| **CD14** | **929** | **1.339656** | **7.38E-06** | **2.138118** | **1.36E-06** | **DEG_up** |
| **CD27** | **939** | **1.481463** | **0.000898** | **3.041468** | **1.03E-10** | **DEG_up** |
| **CD36** | **948** | **-1.3271** | **0.0118** | **-1.30287** | **0.003074** | **DEG_down** |
| **CD38** | **952** | **1.224977** | **0.001552** | **5.257288** | **1.20E-10** | **DEG_up** |
| **CD79A** | **973** | **1.987946** | **0.003982** | **4.51349** | **1.40E-12** | **DEG_up** |
| **CSF3R** | **1441** | **1.095235** | **0.006499** | **1.379434** | **1.74E-05** | **DEG_up** |
| **CXCL1** | **2919** | **1.322065** | **0.043273** | **2.746782** | **5.22E-06** | **DEG_up** |
| **CXCL8** | **3576** | **2.004934** | **0.000564** | **2.5216** | **3.28E-05** | **DEG_up** |
| **CXCR1** | **3577** | **1.226819** | **0.004609** | **3.525905** | **2.98E-07** | **DEG_up** |
| **CXCR4** | **7852** | **1.445889** | **0.000123** | **3.995914** | **4.09E-07** | **DEG_up** |
| **CYBB** | **1536** | **1.21067** | **0.000171** | **1.906909** | **5.81E-06** | **DEG_up** |
| **DCC** | **1630** | **1.097135** | **0.000636** | **1.063572** | **0.000421** | **DEG_up** |
| **DLG2** | **1740** | **-1.11018** | **0.000261** | **-3.24753** | **1.17E-08** | **DEG_down** |
| **ENPP2** | **5168** | **1.251261** | **0.000223** | **1.540053** | **2.41E-06** | **DEG_up** |
| **FCGR2A** | **2212** | **1.262149** | **0.000199** | **3.112776** | **8.94E-11** | **DEG_up** |
| **FCGR2B** | **2213** | **1.078285** | **0.00012** | **3.107189** | **1.08E-10** | **DEG_up** |
| **FCGR3A** | **2214** | **1.282573** | **0.000214** | **2.939193** | **7.47E-10** | **DEG_up** |
| **GPR65** | **8477** | **1.065676** | **0.000168** | **2.503424** | **6.62E-07** | **DEG_up** |
| **IL1B** | **3553** | **1.587251** | **0.000861** | **2.69882** | **2.16E-06** | **DEG_up** |
| **IL6** | **3569** | **1.251642** | **0.013977** | **3.646212** | **0.004131** | **DEG_up** |
| **IRF4** | **3662** | **1.530158** | **0.001031** | **5.660294** | **2.73E-09** | **DEG_up** |
| **MAPT** | **4137** | **-1.83338** | **1.45E-06** | **-1.52342** | **1.01E-06** | **DEG_down** |
| **MERTK** | **10461** | **1.01974** | **2.17E-06** | **1.784187** | **4.06E-06** | **DEG_up** |
| **MMP1** | **4312** | **2.004179** | **0.016386** | **3.649213** | **1.26E-06** | **DEG_up** |
| **MMP3** | **4314** | **1.177284** | **0.023397** | **4.862432** | **9.04E-07** | **DEG_up** |
| **MMP9** | **4318** | **1.042384** | **0.031716** | **3.429994** | **1.32E-05** | **DEG_up** |
| **MS4A1** | **931** | **1.526685** | **0.025156** | **2.271167** | **0.000246** | **DEG_up** |
| **NCF4** | **4689** | **1.01712** | **0.000247** | **2.934114** | **6.90E-11** | **DEG_up** |
| **PIK3CG** | **5294** | **1.041792** | **0.000241** | **3.283468** | **6.03E-09** | **DEG_up** |
| **PIM2** | **11040** | **1.51506** | **0.001442** | **3.301535** | **3.82E-09** | **DEG_up** |
| **POU2AF1** | **5450** | **1.008201** | **0.002618** | **2.688811** | **4.34E-09** | **DEG_up** |
| **PTGS2** | **5743** | **1.154273** | **0.011784** | **1.871367** | **0.034217** | **DEG_up** |
| **PTPN22** | **26191** | **1.124969** | **0.003698** | **2.07864** | **6.66E-08** | **DEG_up** |
| **PTPRC** | **5788** | **1.159485** | **0.001153** | **2.321882** | **2.21E-09** | **DEG_up** |
| **RGS1** | **5996** | **1.588504** | **0.000117** | **3.438155** | **0.003894** | **DEG_up** |
| **SELL** | **6402** | **1.198412** | **0.008616** | **3.321142** | **4.22E-08** | **DEG_up** |
| **SFRP2** | **6423** | **1.00111** | **0.013152** | **1.660797** | **0.000283** | **DEG_up** |
| **SFRP4** | **6424** | **2.715661** | **0.000523** | **2.10257** | **1.64E-05** | **DEG_up** |
| **SPP1** | **6696** | **1.604904** | **0.003732** | **4.436468** | **4.47E-06** | **DEG_up** |
| **ST6GAL1** | **6480** | **1.451207** | **0.000231** | **2.803452** | **2.03E-11** | **DEG_up** |
| **TGM2** | **7052** | **1.126729** | **0.017412** | **3.205605** | **1.60E-07** | **DEG_up** |
| **TLR4** | **7099** | **1.404632** | **1.46E-05** | **1.297981** | **0.001569** | **DEG_up** |
| **TNFRSF17** | **608** | **1.276914** | **0.004377** | **6.433416** | **6.29E-11** | **DEG_up** |
| **TREM1** | **54210** | **1.171852** | **0.000971** | **3.910603** | **5.20E-06** | **DEG_up** |
| **XBP1** | **7494** | **1.131007** | **0.00035** | **3.58848** | **2.63E-11** | **DEG_up** |

**Supplementary Figure 1**: The protein-protein interaction network of periimplantitis (A) and the TF-target network of periimplantitis (B). In the two networks, the size of nodes indicated their higher degree.

**Supplementary Figure 2**: (A) the activated pathway-gene network; (B) the activated TF-target network.
